# Perovskite solar cell for photocatalytic water splitting with a TiO_2_/Co-doped hematite electron transport bilayer

**DOI:** 10.1039/c7ra11996h

**Published:** 2018-01-31

**Authors:** Subhasis Roy, Gerardine G. Botte

**Affiliations:** Center for Electrochemical Engineering Research, Chemical and Biomolecular Engineering Department, Ohio University Athens Ohio 45701 USA botte@ohio.edu

## Abstract

Hydrogen production using a photoelectrochemical (PEC) route promises to be a clean and efficient way of storing solar energy for use in hydrogen-powered fuel cells. Iron oxide (α-Fe_2_O_3_) is best suited to be used as a photoelectrode in PEC cells for solar hydrogen production due to its favorable band gap of ∼2.2 eV. Herein, chemical solution deposition was used for the preparation of a series of Co-doped Fe_2_O_3_ thin films on a titania buffer layer at different doping concentrations (3.0, 7.0 and 10.0 at%). The maximum anodic photocurrent reached up to 3.04 mA cm^−2^ by optimizing the balance between the doping concentrations, enhanced donor density, light absorbance, and surface roughness. The optical properties show that the light absorbance tendency switches to the higher wavelength with the further increment of Co beyond 3.0%. Finally, synthesized photosensitive perovskite CH_3_NH_3_PbI_3_ materials were added as a surface treatment agent on the photoelectrode to enhance the photocurrent absolute value. This inorganic nanostructured perovskite CH_3_NH_3_PbI_3_ (MAPbI_3_) coated on the Co-doped hematite photoanode achieved an overall solar-to-hydrogen conversion efficiency of 2.46%. Due to its low temperature processing, stability, and enhance efficiency, this perovskite coated TiO_2_/Co-doped hematite multilayer thin film solar cell has high potential to be applied in industry for hydrogen production.

## Abstract 2

Performance optimisation of a halide nanostructured perovskite CH_3_NH_3_PbI_3_ (MAPbI_3_) coated cobalt doped hematite photoanode for efficient photocatalytic water splitting.

## Graphical abstract

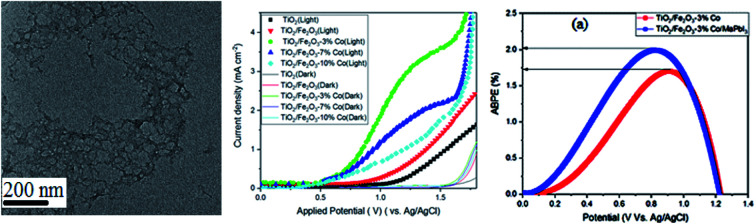

## 1. Introduction

Photoelectrochemical (PEC) water splitting is an encouraging technology in solar hydrogen production for building a renewable and clean energy economy. This solar hydrogen has been demonstrated to be advantageous in storing solar energy in the form of chemical bonds such as in H_2_ through the PEC splitting of water to provide a net supply of reducing equivalents. Hematite (α-Fe_2_O_3_) based photoanodes are ideal for such applications, due to their stability, abundance and low cost.^1,2^ In spite of these encouraging properties, progress towards the manufacture of useful water splitting devices has been limited. The free energy change required to split one molecule of H_2_O to H_2_ and 1/2O_2_ under standard conditions is 237.2 kJ mol^−1^ while the cell voltages are in the order of 1.8–2.0 V.^3,4^ This potential requirement could be attained from a semiconductor photoanode with appropriate valence and conduction bands illuminated by visible light. Such a reaction involves the use of a single semiconductor that absorbs two photons to generate a molecule of H_2_. Nevertheless, designing two photons system that produces carriers of high photopotential, while possessing appropriate energy levels to drive the water splitting reaction has proven challenging. High band gap of the semiconductor to generate a larger photopotential results in poorer solar absorption and less photocurrents.^5–7^ These energetic limitations along with the critical stability problem have not been efficiently encountered by a single photoanode semiconductor based system. However designing a system for efficient water splitting, the choice of the photoelectrode is a serious issue. Hematite (Fe_2_O_3_) is a stable semiconductor photoanode, economically viable with an appropriate band gap for catalyzing the oxygen evolution reaction using visible light. The maximum theoretical solar-to-hydrogen conversion efficiency of hematite is predicted to be 16.8%.^8^ Though, the kinetics of hole injection and the short hole diffusion lengths limit the performance of hematite. Also, the conduction band position is not favorable for H_2_ reduction demanding an external bias to drive the complete water splitting reaction. Choosing solar catalyst materials that can drive the hematite photoelectrode for water splitting requires two criteria to be satisfied sufficient spectral mismatch and a significant photovoltage.^9^ Since Fe_2_O_3_ has a band gap of 2.1 eV, the semiconductor utilized in the solar cell should possess a smaller band gap for absorption in the solar spectrum and still be able to deliver enough photovoltage.

In this study, we report a simple method to produce mesoporous Co doped Fe_2_O_3_–TiO_2_ bilayer films that have a suitable valence band structure to photo-oxidize water to H_2_ while having a band gap covering the entire range of visible light. It is known from the literature that titanium dioxide (TiO_2_) is thermally stable, non-flammable, poorly soluble, and not classified as hazardous.^9^ TiO_2_ thin films have been developed for applications that require high resistance transparent (HRT) buffer layers (BL) to reduce recombination losses. TiO_2_ thin films also have excellent photocatalytic properties.^9^ Co doped Fe_2_O_3_–TiO_2_ films exhibit a heterostructure depending on the Co content that limits the e^−^/h^+^ recombination. TiO_2_ compounds containing Fe^3+^ were reported to have a band gap of 2.3 eV and exhibit a p–n junction.^10,11^ Environmental purification using doped α-Fe_2_O_3_ photocatalyst has attracted a great deal of attention with the increasing number of recent environmental problems in the world. Doped α-Fe_2_O_3_ photoactive thin film has excellent photocatalytic properties as well as high transparency, excellent mechanical and chemical durability. Theoretically this film can reach maximum solar to hydrogen conversion efficiency with very low cost.^10,11^ Owing to this feature, TiO_2_ containing Fe^3+^ ions having a band gap energy of 2.3 eV have been reported as photo-catalysts. However, to the best of our knowledge, none of this Co doped Fe_2_O_3_–TiO_2_ mesoporous films have been investigated for solar energy conversion. We demonstrate that a single inorganic halide perovskite (CH_3_NH_3_PbI_3_) coated Co doped Fe_2_O_3_–TiO_2_ solar cell can fulfil these requirements and be utilized to drive photoelectrochemical water splitting. To overcome the stability issue of MAPbI_3_ coated photoanode in water atmosphere, aqueous hydroiodic acid solution was used as per literature.^12^ This study for the first time highlights a sol–gel chemistry cost effective synthesis route of new nanoheterostructured mesoporous materials with a large interfacial area for solar hydrogen production.

## 2. Experimental

### 2.1 Perovskite crystals synthesis

CH_3_NH_3_PbI_3_ (MAPbI_3_) was synthesized according to reported procedures with some modifications.^13,14^ 0.3 mol (38 mL) of methylamine (CH_3_NH_2_) (33 wt% in absolute ethanol, Sigma Aldrich, anhydrous, ≥98%) was allowed to react with equimolar (40 mL) hydroiodic acid (HI) (57 wt% in water, Sigma Aldrich, anhydrous, ≥99%). The reaction involved stirring at 500 rpm for 30 seconds on a stirring plate (Corning PC-620 D using a 1′′ stir bar) at 0 °C for about 2 h to synthesize methyl ammonium iodide (CH_3_NH_3_I). A bath of ice and water maintain a temperature of 0 °C with addition of sodium chloride to the ice (1 to 3 ratio by weight). To achieve crystallized form of CH_3_NH_3_I, evaporation was carried out at 60 °C on a heating plate (Corning PC-620 D) for 2–3 hours using 1′′ stir bar at 200 rpm. Crystallized CH_3_NH_3_I powder along with equimolar lead(ii) iodide (PbI_2_) (Alfa Aesar, 98.5% purity) was dissolved in ethanol (anhydrous, Fisher chemical, 95.27%) under stirring at 60 °C for 3 h at 500 rpm on a stirring a plate (Corning PC-620 D) using 1′′ stir bar to produce CH_3_NH_3_PbI_3_ precursor solution. Final produced 10 mL solution was preserved in a sterile plastic tube (Fisher brand).

### 2.2 Fabrication of photoelectrodes

**Fig. 1:**
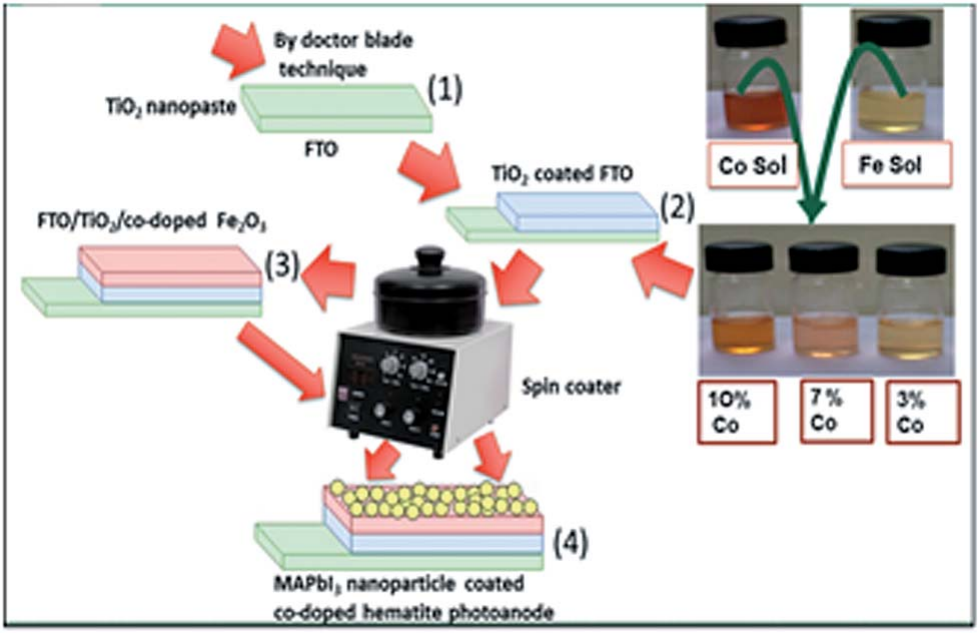
Schematic flowsheet perovskite based FTO/TiO_2_/Co-doped hematite thin films for photocatalytic water splitting.

Fig. 1 shows the schematic flowsheet for the synthesis of perovskite based FTO/TiO_2_/Co-doped hematite thin films for photocatalytic water splitting. The TiO_2_ film was prepared by doctor-blade method on fluorine-doped tin oxide (FTO) (10 mm × 20 mm × 2.2 mm) glass substrate (15 ohms sq^−1^, Techinstro) using commercial titanium dioxide nanopaste (Solaronix, ∼18% wt). This glass substrate was pre-cleaned with a detergent solution (Great Value Glass Cleaner 32oz), deionized (DI) water, and ethanol in an ultrasonic bath (Zenith) at 50 kHz for 2 min to remove trap dirt and dust. The coated titanium monolayer which acts as a buffer blocking layer was inserted into a preheated furnace (MTI Corporation OTF-1200X) and kept at 500 °C for 30 min for organic removal and cooled to room temperature. Next cobalt doped hematite thin films were deposited on the titanium oxide layer by spin coating (CHEMAT Technology spin coater KW-4A) 0.15 M precursor solution, prepared by dissolving Fe(NO_3_)_3_·9H_2_O (ACROS Organics, 98%) with varying dopant concentrations (0, 3.0, 7.0 and 10.0 at% volume) of Co(NO_3_)_3_·9H_2_O (ACROS Organics, 99%, pure) in DI water. The spin coater speed was kept at 3000 rpm for 30 seconds and the produced film was heated in a heating plate (Corning PC-620 D) for 5 min at 150 °C. The coating and heating sequence were repeated 10 times to attain films of about 400 nm thick. After the final coating, films were sintered at 500 °C for 2 h in air in a furnace (MTI Corporation OTF-1200X) for crystallization followed by natural cooling. Finally synthesized MAPbI_3_ nanocrystals were spin coated at 2500 rpm for 30 s and heated for 5 min at 120 °C. The coating and firing sequence were repeated 5 times. This coated glass was place in open atmosphere for 1 h to attain normal condition. Finally, ohmic electrical contacts for PEC measurements were generated using silver conductive adhesive paste (Alfa Aesar) and a copper wire loop (Good fellow copper-wire, diameter: 0.125 mm, purity 99.95%).

### 2.3 Characterization of materials

The crystal structures and the grain size of the prepared samples were determined using X-ray diffraction (XRD, Ultima III, Rigaku, Japan) with Cu Kα (*λ* = 1.5405 Å) radiation operated at 40 kV and 30 mA. Energy dispersive X-ray spectroscopy (EDS) (HRTEM: JEM-2100, JEOL, Japan) was used to determine the elemental composition of the material. The microstructural properties and crystal structures of the samples were characterized using a High Resolution Transmission Electron Microscope (HRTEM) system (HRTEM: JEM-2100, JEOL, Japan) operated at 200 kV. The optical absorption of thin films on FTO glass was examined by UV-spectrophotometer (Agilent Technology-8452A) applying 200–800 nm length region. Finally, photoelectrochemical properties of hematite/perovskite photoanodes were evaluated in a homemade 2 neck beaker using a Solartron potentiostat (SI-1287A). A three-electrode configuration was employed, with a Pt counter electrode (size 1′′ × 1′′, 99.99% metals basis, Alfa Aesar) and Ag/AgCl/sat. KCl reference electrode. In the case of Co-doped Fe_2_O_3_, the electrolyte consists of 0.5 M Na_2_CO_3_ (Sigma Aldrich, reagent grade, 99.5%) in water which gives a pH of 11.2. In the case of MAPbI_3_ coated Co-doped Fe_2_O_3_ photoelectrode PEC, the electrolyte used consisted of synthesized MAPbI_3_ powder and 6.06 M aqueous HI as per the recent literature.^7^ The sample was irradiated at the electrolyte/semiconductor interface and the illuminated area was determined to be 0.8 cm^2^. Potentials were applied *versus* the Ag/AgCl reference electrode and illumination was provided by a white LEDs (with an intensity of 25 mW cm^−2^), placed 12.5 cm in front of the sample. Illumination power was controlled by applying a fixed current to the LEDs.

## 3. Results and discussion

**Fig. 2:**
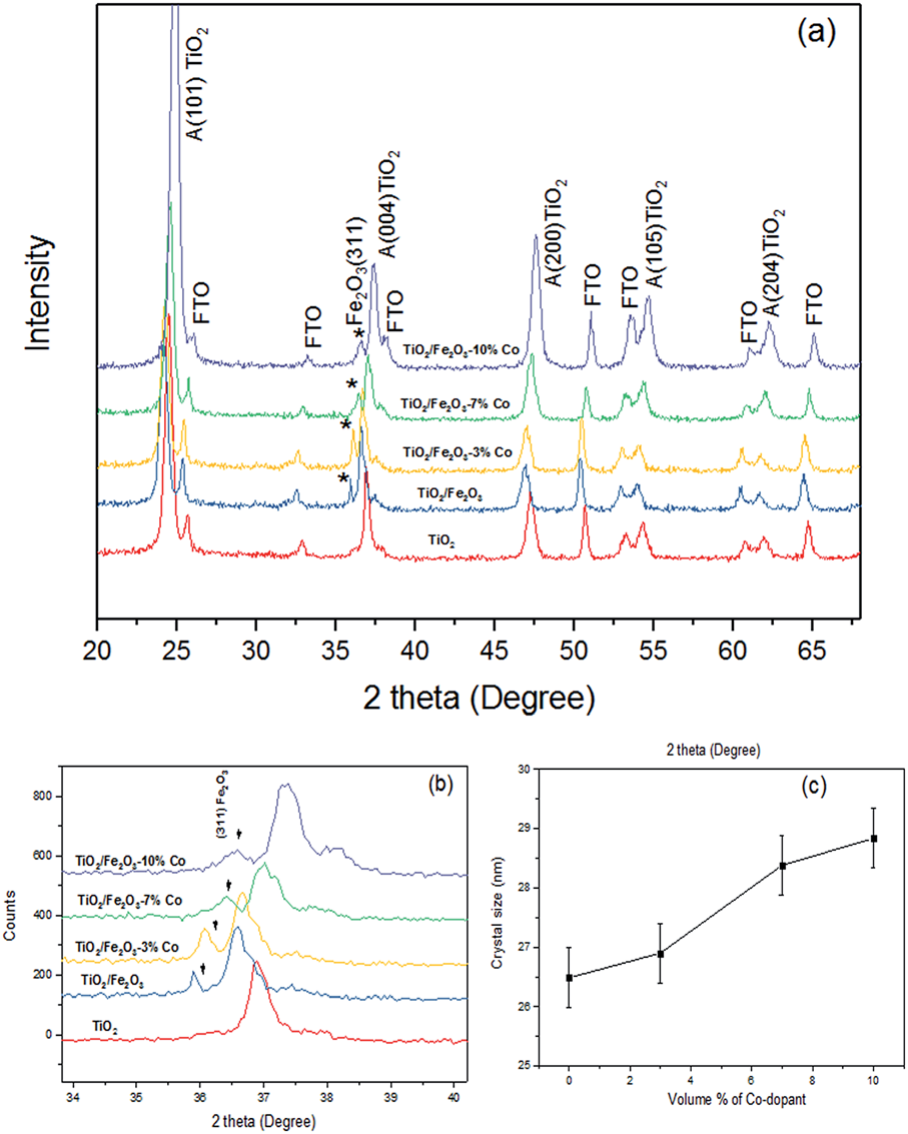
X-ray diffraction pattern of (a) TiO_2_, undoped Fe_2_O_3_, 3% Co-doped Fe_2_O_3_, 7% Co-doped Fe_2_O_3_ and 10% Co-doped Fe_2_O_3_ thin films. (b) Magnified diffraction peak of Fe_2_O_3_ at (311) direction plane with varying dopant concentration (c) average basal size of the grown hematite as a function of doping, estimated from the width of the (311) Bragg peak.

Fig. 2 illustrates the XRD patterns of undoped and 3, 7 and 10% Co-doped films of Fe_2_O_3_ on titania. All the samples of doped/undoped hematite were prepared by chemical solution deposition method. As shown in Fig. 2a, the most intense dominating peaks are titanium dioxide and FTO substrate. A weak diffraction peak at 35.8° (star symbol in Fig. 2a and b) is only identified which indicates that the pure hematite and all Co doped hematite nanoparticle growth orientation are along the [110] direction according to the JCPDS card no. 033-0664. The intensity of this strongest peak tends to decrease and its line width broadens as the Co dopant concentration increases reflecting a change of texture. Peaks shifted slightly to higher angles with increasing Co dopant. This was attributed to the lattice expansion due to the substitution of Fe^3+^ by Co^2+^, because the ionic radius of Co^2+^ (0.74 Å) is higher than the ionic radius of Fe^3+^ (0.64 Å). The other peaks in Fig. 2 indicates the Bragg position for FTO layer and anatase TiO_2_ according to the JCPDS card no. 041-1445 and JCPDS 75-1537, respectively. The higher intensity observed in the three samples with smaller doping is an indication that a major set of planes oriented in the direction [311] (Fig. 2b) are contributing to the signal in the diffractogram, which evidences that the crystallinity of the samples improves with low Co^2+^ contents during the synthesis process.^15^ There is no evidence of XRD reflections from Co, which confirms the purity of the doped α-Fe_2_O_3_. The average particle size calculated from XRD data using Scherrer's equation^16^ varies from 26–29 nm (Fig. 2c), while this value rises for the higher doping cobalt in hematite samples.

**Fig. 3:**
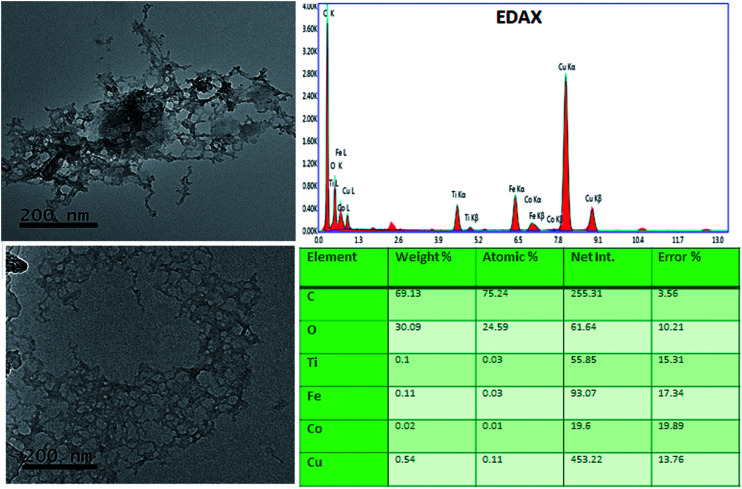
High resolution transmission electron microscopy images of titania/Co-doped hematite thin films with EDX spectrum.

High-resolution transmission electron microscope (JEOL JEM-2100F) was used to investigate the uniformity and particle size distribution of the sample. TEM images (Fig. 3) reveals porous connecting nanoparticles with average crystallite size is close to 30–50 nm and surface area of the pore diameter varies from 20–50 nm. Elemental analysis was determined by mapping TEM grid employing TEM-EDAX measurement. The data clearly confirm the presence of all the constituents including Co, Fe, Ti, O. Signatures of Cu and C appeared for carbon coated cupper grid substrate.

**Fig. 4:**
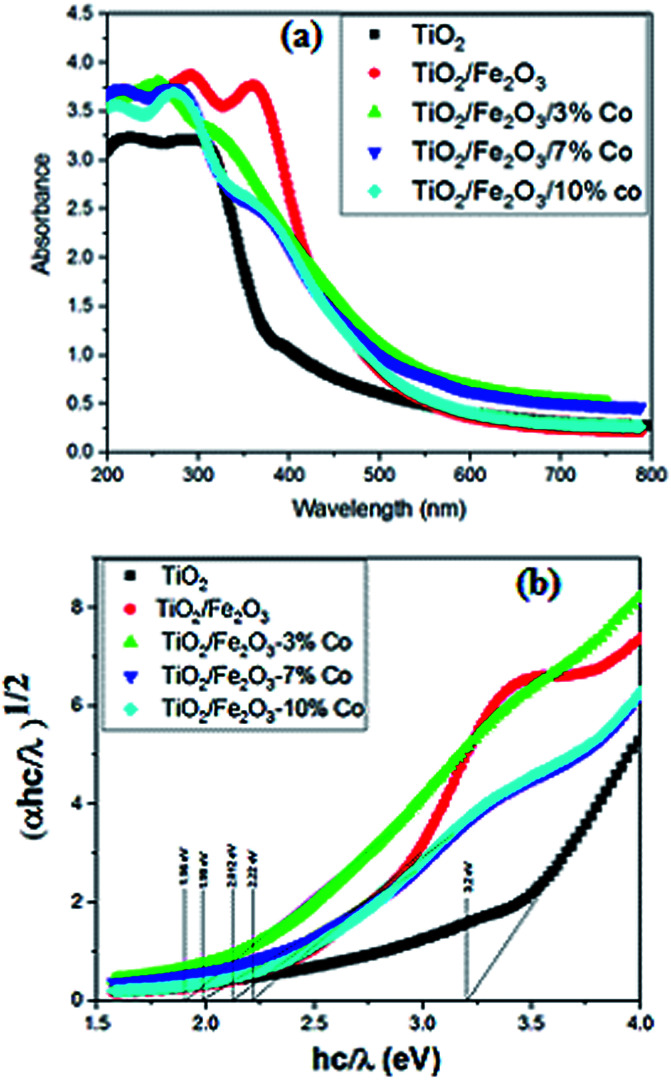
(a) Absorbance curve (b) Tauc plot for all titania/Co-doped hematite thin films.

The optical density (or absorbance measured by Agilent Technology-8452A spectrometer) spectrum of the films is shown in Fig. 4a. It was found that the highly transparent TiO_2_ might not affect the optical characteristics and behaviour of the TiO_2_/Fe_2_O_3_ films as compared to the Fe_2_O_3_ films.^17^ Absorbance rapidly increased with the wavelength lower than 600 nm, assigned to the electron–hole pair excitation in TiO_2_, Fe_2_O_3_ and cobalt doped Fe_2_O_3_ under ultraviolet and visible region. Then, the absorbance slightly decreased at the wavelength over 600 nm, this is considered to be the result of the charge transfer transition.^18^

Moreover, the absorbance of the films at a wavelength over 600 nm increased with the cobalt dopant concentration and the 3% Co–Fe_2_O_3_/TiO_2_ film shows the highest absorption spectra compared with the films with higher Co dopant content in hematite. This is because the doping effect can improve the crystal structures which can lead to the absorption ability increased in the visible light region. Although it is also natural that the film thickness affect the absorption property of the prepared film. Band gap energies were calculated using the following equation

1 (*αhν*) = *A*(*hν* − *E*_g_)^*n*^

where *α* is the absorption coefficient, *A* is a constant related to the effective mass of the electrons and holes, *E*_g_ is the energy gap and *n* is a constant related to the type of optical transition, *n* = 2 for indirect transition and *n* = 1/2 for the direct transition. Titania and hematite both have been suggested to have a direct band gap.^19^ However, an indirect band gap for hematite is also reported.^20^ Within this wavelength range, the (*αhν*)^1/2^*vs.* (*hν*) plot (Tauc plot) is linear (shown by solid line in Fig. 4b), indicating the direct nature of the band gap. This linear trend was extrapolated (dotted line) to the *hν* axis to calculate the band gap. When the Co doping percentage was increased, the reduction in band gap energy from the initial 2.22 eV (0% Co) to final 1.96 eV (3.0% Co) was observed and improved the minimum energy required for excitation electron.

The electron will become easily excited from the valence band to the conduction band. It was observed that the band gap increased with increasing dopant percentage. This reason might be due to growth process when some impurity (oxygen vacancies and/or Fe interstitials, *etc.*) levels form the edge of the conduction band and these levels can inoculate with the conduction band when the thickness increased. Also, there is a possibility of structural defect in the films due to their preparation at room temperature; this could give rise to the allowed states near the conduction band in the forbidden region. The values of the width related to the bands of hematite nanoparticles which are synthesized with different bases range between 2.22 and 1.96 eV. They are in perfect agreement with the values reported in the literature.^21^

**Fig. 5:**
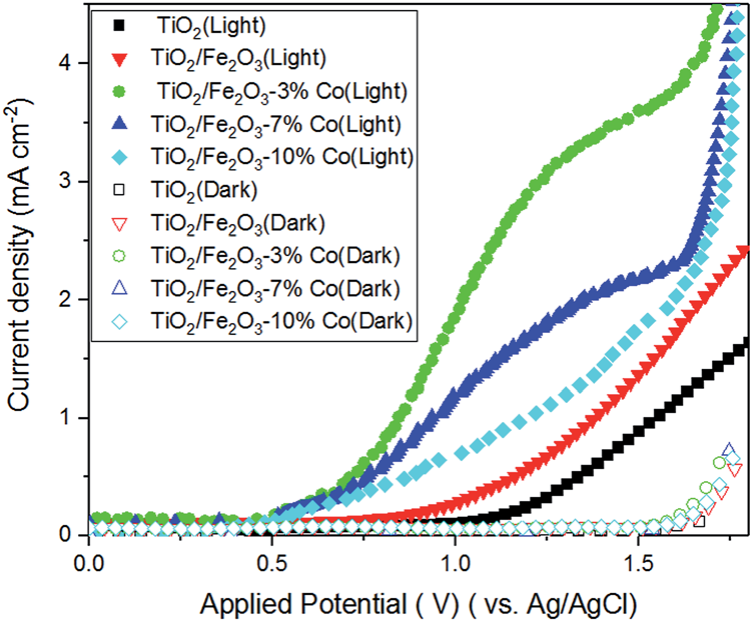
Current density *vs.* applied potential (*J*–*V*) plots for the Co doped Fe_2_O_3_–TiO_2_ electrodes as a function of the Co content under visible light illumination.

Fig. 5 shows the PEC characteristics of the titania–hematite electrode under dark and with 25 mW cm^−2^ illumination. Solution of Na_2_CO_3_ (11 pH) was used as an electrolyte and potentiostat (Solartron SI-1287A) was used for PEC measurement. The photoanode performance is consistent with other studies employing this type of nanostructured hematite.^22,23^ The photocurrent is considered negligible at applied potentials below 0.8–0.9 V *vs.* reference electrode, which defines the onset potential. As the applied potential is increased anodically, the photocurrent exhibits a sharp rise and saturates from a potential of 1.6 V *vs.* reference electrode. The photocurrent plateau, measured at 1.23 V *vs.* reference electrode was found to be linear with the illumination intensity. The flat band potential was determined for these nanostructured photoanodes to be between 0.3 and 0.5 V *vs.* reference electrode in the same electrolyte^22^ considering the onset potential at 0.8–0.9 V *vs.* reference hydrogen electrode as observed in Fig. 5. This demonstrates the typical loss of 0.5 V required for the water splitting reaction to being initiated on hematite electrodes. Previous studies have indicated that applied bias is required to reduce bulk electron–hole recombination, promoting water oxidation on the surface of hematite.^23^ Herein we quantitatively elucidate the effect of recombination at low applied potentials. Photocurrent densities as calculated from the *I*–*V* data which are observed to increase with increasing doping concentration and at 3.0% Co doping offered the best photocurrent density. It seems that the 3.0 at% Co doping in Fe_2_O_3_ is the optimal concentration above which (for 7.0 at% Co) the observed photocurrent density decreases. It is also indicating a high internal quantum efficiency *i.e.*, high charge transport and charge transfer efficiencies in the Co doped hematite photoelectrode. Therefore, this comparison indicates that the higher electric conductivity of 3.0% Co doping hematite, comparing to pure Fe_2_O_3_ or TiO_2_ thin film, enables to improve the charge transport properties and which in turn minimizes the charge carrier's recombination and finally achieves an excellent PEC performance that is comparable with the theoretically expected value.

**Fig. 6:**
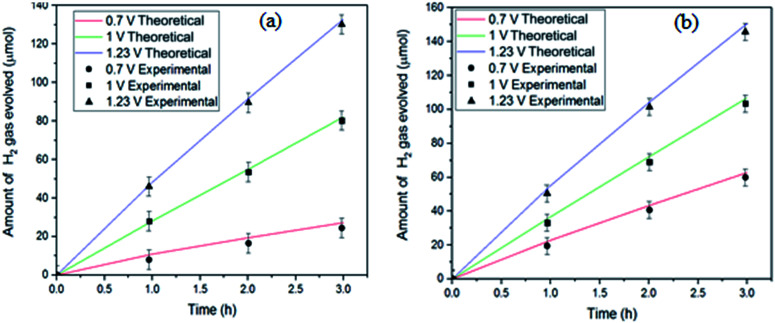
Faradaic efficiency measurement for device (a) bare 3% Co–Fe_2_O_3_/TiO_2_ and (b) MAPbI_3_ coated 3% Co–Fe_2_O_3_/TiO_2_ photoanode by gas chromatography.

**Fig. 7:**
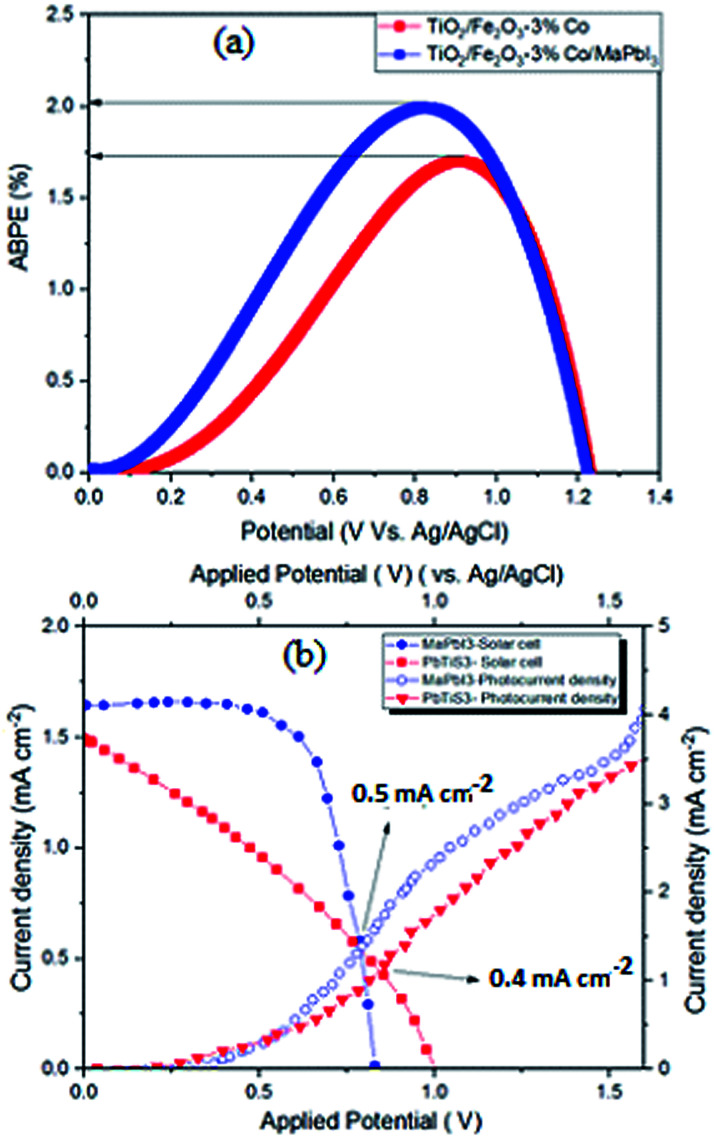
(a) ABPE obtained using a three-electrode system for 3% Co–Fe_2_O_3_/TiO_2_ before and after surface treatment with perovskite MAPbI_3_ and (b) STH efficiency obtained from current density–voltage curve of the perovskite solar cell–hematite photoanode tandem devices together.

In order to estimate the faradaic efficiency and the stability of the tandem device, we performed gas evolution experiments (H_2_) in the PEC reactor under 25 mW cm^−2^ irradiation in the bilayer tandem configuration. These experiments confirm that the generated photocurrent is translated to H_2_ at the respective electrodes. The evolved gases from the tandem configuration were measured continuously for up to 3 h. The amount of evolved H_2_ with time is presented in Fig. 6. The theoretical amounts of evolved gases were calculated by integrating the photocurrent with time while considering that the H_2_ evolution reactions are a two-electron process (straight lines in Fig. 6). On the basis of the amount of H_2_ evolved with time, the calculated faradaic efficiency was 95% for our tandem device. The 5% mismatch in faradaic efficiency might be due to the manual sampling error. Long-term measurements of up to 3 h were done; however, due to the ambient humidity conditions (RH of 60–70%), the encapsulated perovskite solar cells decayed in performance and the photocurrent of the tandem cell dropped to the low percentage. The applied bias photon-to-current efficiency (ABPE) of the 3% Co–Fe_2_O_3_/TiO_2_ electrode calculated by using its *J*–*V* curve and 95% faradaic efficiency at 25 mW cm^−2^ irradiation, is plotted in Fig. 7. The maximum applied bias photon-to-current efficiency achieved for MAPbI_3_ was 2.04%, which is much higher than that of pure Co-doped hematite photoanode (1.72%).

Moreover, this efficiency is achieved at a potential as low as 0.8 V *vs.* reference Ag/AgCl electrode which is a highly favourable feature for a photoelectrochemical diode. It is well-known that the stability of photoanode is another important property for the PEC water splitting. The moderate stability of MAPbI_3_/3% Co–Fe_2_O_3_/TiO_2_ was tested by obtaining a *J*–*t* curve. A photocurrent density of 2.83 mA cm^−2^, obtained by applying 1.23 V between the working and counter electrodes, was maintained for 5 hours irradiation without additional sacrificial reagent. There is not obvious decay of photocurrent density for the samples, which indicates their stability.

Solar-to-hydrogen (STH) efficiencies can be expressed as the fraction of incident solar energy photo-converted into chemical energy:

2 STH = *J*_SC_*E*^0^*η*/*P*_total_

where *J*_SC_ is the short-circuit current, *E*^0^ corresponds to the thermodynamic reaction potential solar-to-hydrogen (STH) estimated using large area devices (1 × 0.8 cm^2^).^24^ The operating current densities of the separate components and together in the cell were calculated by the crossing point of two photocurrent densities curves. The value of the operating current density in current density–voltage curve of the perovskite solar cell and hematite photoanode tandem devices measurements are 0.5 mA cm^−2^ and 0.4 mA cm^−2^ for MAPbI_3_/Co doped hematite, respectively. The small inconsistency between these two measurements can be ascribed to the losses inside the PEC chamber and electrolyte. The STH efficiencies were calculated to be 2.46% for MAPbI_3_ coated Co doped hematite thin film at 25 mW cm^−2^ irradiation (see Fig. 7b).

In this hybrid bilayer film, TiO_2_ acts as a buffer layer for minimizing the presence of recombination centers. Additionally, Co-doped α-Fe_2_O_3_ exhibit less localized features signifying a smaller effective electron mass which improved electron conductivity when compared with the pure α-Fe_2_O_3_. As a result, the charge carrier density is greatly improved, and due to fewer recombination sites, Co-doped α-Fe_2_O exhibit enhanced photoactivity, in agreement with our experimental observations.^25^ Also it can be predicted that Co-doped α-Fe_2_O_3_ photoanodes exhibited lower electron transport resistances compared to undoped samples, consistent with the enhanced electrical conductivity. Since the optimized structures are obtained by relaxing only the atomic coordinates, large formation energies imply not only low dopant concentration, but also reduced structural stability. So here we optimized the Co dopant concentration level in α-Fe_2_O_3_ to enhance high water splitting response.

The unique mesoporous structure of Co-doped α-Fe_2_O_3_ facilitates effective photon to electron interaction due to high surface area. The properties of mesoporous, including high mean free path, pore size, and porosity as well as the surface properties, can be altered depending on dopant level. Active surface permits functionalization to change surface properties. Simple bilayer structures films do not favor separation of photogenerated charge carrier. Transfer mechanism and charge separation is also restricted in this type of films.

## 4. Conclusions

The present work focused on improving the performance (photocurrent density and stability) of hematite photoanodes. We have successfully employed a multi-layer sol–gel technique for the synthesis of mesoporous nanostructured Co doped Fe_2_O_3_–TiO_2_ bilayer films with tuneable composition (*x*). These films are constituted of well-defined nanocrystallites, with small size and an interconnected network of pores and display the properties required for photoelectrochemical applications through a highly controllable process and at a relatively low processing temperature. This deposited film has high specific surface area to reach high interface with the electrolyte. This film also maintained optical transparency in the visible region and high densities of electroactive components, numerous solid/solid interfaces that stabilize the hole and as a consequence avoid the e^−^/h^+^ recombination in the bulk. This optimized 3% Co-doped hematite photoanodes significantly improve overall solar-photon to photocurrent efficiency at very low cost as hematite is inexpensive and low processing temperature. This hybrid hematite films with porous nanostructure enabled high photon harvesting efficiency and maximized interfacial charge transfer. This photoanode film can be useful as a catalyst for organic photo-degradation and as well as for water splitting H_2_ generation. Finally, after surface treatment, inorganic halide nanostructured perovskite (CH_3_NH_3_PbI_3_) coated hematite photoanode achieved overall solar-to-hydrogen conversion efficiency of 2.46%. This is a significant improvement in the state-of-the-art of hematite photo anodes through an enhancement in electron transport in films by decreasing over potential. Due to the low processing temperature, stability, and enhance efficiency the use of this hematite film represents a promising design for the development of new architectures for the next technologies in artificial water splitting.

## Conflicts of interest

There are no conflicts to declare.

## Supplementary Material
